# Early cerebral amyloid-*β* accumulation and hypermetabolism are associated with subtle cognitive deficits before accelerated cerebral atrophy

**DOI:** 10.1007/s11357-023-01031-w

**Published:** 2023-12-16

**Authors:** Aftab Bakhtiari, Krisztina Benedek, Ian Law, Birgitte Fagerlund, Erik Lykke Mortensen, Merete Osler, Martin Lauritzen, Henrik B. W. Larsson, Mark B. Vestergaard

**Affiliations:** 1 Functional Imaging Unit, Department of Clinical Physiology and Nuclear Medicine, Rigshospitalet Glostrup, Rigshospitalet, University of Copenhagen, Copenhagen, Denmark; 2 Department of Clinical Neurophysiology, The Neuroscience Centre, Rigshospitalet, University of Copenhagen, Copenhagen, Denmark; 3 Department of Clinical Physiology and Nuclear Medicine, Rigshospitalet, , University of Copenhagen, Copenhagen, Denmark; 4 Faculty of Health and Medical Sciences, Department of Neuroscience, University of Copenhagen, Copenhagen, Denmark; 5 Center for Healthy Aging, Faculty of Health and Medical Sciences, University of Copenhagen, Copenhagen, Denmark; 6 Department of Neurology, Neurophysiology, Zealand University Hospital, Roskilde, Denmark; 7 Department of Psychology, University of Copenhagen, Copenhagen, Denmark; 8 Child and Adolescent Mental Health Center, Copenhagen University Hospital – Mental Health Services CPH, Copenhagen, Denmark; 9 Department of Public Health, University of Copenhagen, Copenhagen, Denmark; 10 Center for Clinical Research and Prevention, Bispebjerg and Frederiksberg Hospital, Copenhagen, Denmark; 11 Faculty of Health and Medical Sciences, Department of Clinical Medicine, University of Copenhagen, Copenhagen, Denmark

**Keywords:** Aging, Cognition, Atrophy, Amyloid, Cerebral glucose metabolism, PET imaging

## Abstract

**Aims:**

Alzheimer’s disease (AD) is characterized by the accumulation of amyloid beta (A*β*) in the brain. The deposition of A*β* is believed to initiate a detrimental cascade, including cerebral hypometabolism, accelerated brain atrophy, and cognitive problems—ultimately resulting in AD. However, the timing and causality of the cascade resulting in AD are not yet fully established. Therefore, we examined whether early A*β* accumulation affects cerebral glucose metabolism, atrophy rate, and age-related cognitive decline before the onset of neurodegenerative disease.

**Methods:**

Participants from the Metropolit 1953 Danish Male Birth Cohort underwent brain positron emission tomography (PET) imaging using the radiotracers [^11^C]Pittsburgh Compound-B (PiB) (*N* = 70) and [^18^F]Fluorodeoxyglucose (FDG) (*N* = 76) to assess cerebral A*β* accumulation and glucose metabolism, respectively. The atrophy rate was calculated from anatomical magnetic resonance imaging (MRI) scans conducted presently and 10 years ago. Cognitive decline was examined from neurophysiological tests conducted presently and ten or 5 years ago.

**Results:**

Higher A*β* accumulation in AD-critical brain regions correlated with greater visual memory decline (*p* = 0.023). A*β* accumulation did not correlate with brain atrophy rates. Increased cerebral glucose metabolism in AD-susceptible regions correlated with worse verbal memory performance (*p* = 0.040).

**Conclusions:**

A*β* accumulation in known AD-related areas was associated with subtle cognitive deficits. The association was observed before hypometabolism or accelerated brain atrophy, suggesting that A*β* accumulation is involved early in age-related cognitive dysfunction. The association between hypermetabolism and worse memory performance may be due to early compensatory mechanisms adapting for malfunctioning neurons by increasing metabolism.

**Supplementary Information:**

The online version contains supplementary material available at 10.1007/s11357-023-01031-w.

## Introduction

Increasing age is associated with structural and functional brain alterations, including atrophy, amyloid beta (A*β*) deposition, and reduced cerebral glucose metabolism. The latter are particularly interesting since they constitute early biomarkers for diagnosing and predicting conversion from healthy aging to mild cognitive impairment (MCI) and Alzheimer’s disease (AD)—especially when combined [1]. One of the first pathogenic components of Alzheimer’s disease (AD) is believed to be the deposition of aggregated A*β* in the brain [2] and elevated A*β* deposition is also observed in patients with mild cognitive impairment (MCI) before conversion [3]. As such, A*β* deposition is an important biomarker for diagnosing AD and identifying prodromal or preclinical AD. Although A*β* deposition alone is insufficient to diagnose AD, A*β*-positive individuals without cognitive impairment are regarded as asymptomatic at risk [4]. However, the progression rate for asymptomatic A*β*-positive individuals to AD is uncertain. An important factor in differentiating prodromal AD is based on the regional deposition of A*β*. In AD, A*β* deposition typically first occurs in the cingulate gyrus, precuneus, orbitofrontal cortex, and temporal lobe, with subsequent spreading to the prefrontal and parietal cortices [5]. A*β* deposition appears to occur in the same areas in normal aging, albeit with less intensity and less involvement of the posterior cingulate and precuneus [6, 7]. The degree of A*β* burden is also affected by genotype, where apolipoprotein E epsilon 4 (APOE-*ε*4) positive individuals may develop significant A*β* deposition at an earlier age and experience a more rapid accumulation [8]. A*β* deposition can be measured in-vivo with positron emission tomography (PET) using the [^11^C]Pittsburgh Compound-B (PiB) tracer, which binds to A*β* proteins in the brain [9].

The brain has a high energy consumption and requires approximately 20% of all glucose-derived energy [10]. Most of this energy consumption is used to maintain neuronal and synaptic functioning, such as action/postsynaptic/resting potentials and biosynthesis of neurotransmitters [10]. Reduced cerebral glucose metabolism occurs in healthy aging and is further accelerated in neurodegenerative diseases such as MCI and AD. Brain glucose metabolism can be measured regionally using [^18^F]Fluorodeoxyglucose PET (FDG-PET), and can therefore be used to determine hypometabolism associated with neurodegenerative disease. FDG-PET hypometabolism is thought to reflect neuronal dysfunction and cortical atrophy and is, therefore, characterized as a marker of neurodegeneration [1]. Reduced cerebral glucose consumption is also observed in normal healthy aging, mainly in the anterior cingulate, frontal, and temporoparietal areas [11, 12]. In AD, hypometabolism is initially observed in the parieto-temporal cortices, the posterior cingulate cortex, and the precuneus, followed by the frontal cortex in the later stages of the disease [1, 13].

AD pathophysiology likely begins 10–20 years before clinical symptoms emerge [14], and evolves following a temporal order, beginning with A*β* accumulation, followed by tau aggregation, hypometabolism, brain atrophy, and cognitive decline, before ultimately, clinical symptoms emerge [15]. Furthermore, all biomarkers are postulated to become progressively worse parallel to disease progression. However, it is not fully understood which pathological cascades are initiated first from A*β* deposition or whether A*β* alone is sufficient to affect cognitive functions. The present study examined how A*β* deposition correlates with cognitive decline, decennial brain atrophy rate, and cerebral glucose metabolism in a cohort of cognitively normal subjects aged 66–68, which we have followed for the last 10 years. Using this setup and cohort, we could examine the early effects of A*β* deposition in subjects before potential dementia diseases. Based on the amyloid cascade hypothesis and the dynamic-biomarker model [15–17], we hypothesize that increased PiB is associated with worse cognitive performance, higher decennial atrophy rate, and reduced FDG. Moreover, we hypothesize that decreased FDG is also correlated with worse cognitive performance. We believe this study, which has employed a birth cohort consisting of a homogenous group with longitudinal neuropsychological and neuroimaging measurements, constitutes a unique dataset with the potential to provide important insight into the temporal development of AD pathophysiology.

## Methods

### Participants

**Fig. 1:**
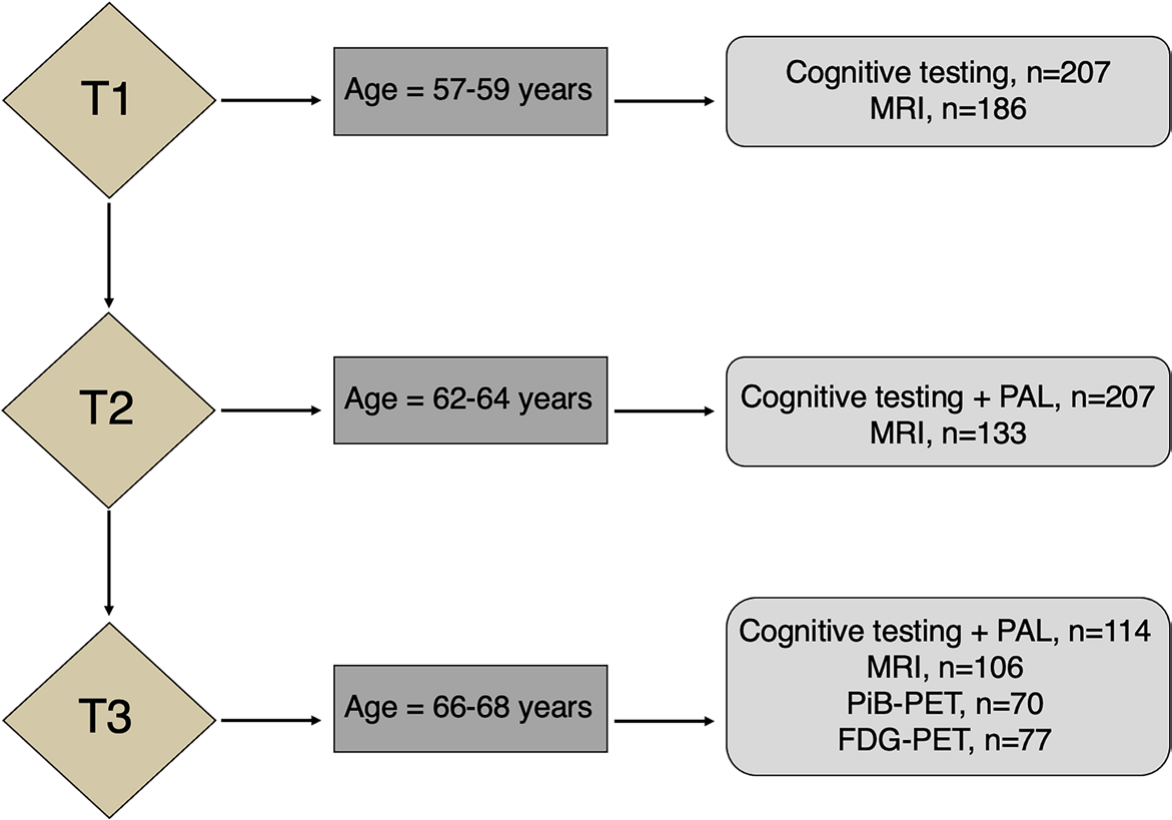
Flowchart illustrating experiment setup, including age of participants at each timepoint and each included imaging modality

Participants were recruited from The Metropolit 1953 Danish Male Birth Cohort, established in 1965 and defined as all boys born in 1953 in the Copenhagen Metropolitan area [18]. The participants have been examined multiple times throughout their lives concerning physiological state and cognitive skills. These longitudinal assessments make the cohort optimal for assessing age-related changes in brain function from a life course perspective. From the same subset of participants, we have previously found that compromised blood–brain barrier permeability is associated with increased vascular risk factors [19] and associations between cognitive dysfunction and electrophysiological changes [20]. Previously published work on the cohort has also reported associations between cognitive dysfunction and cerebrovascular functions [21]. For the current study, we examined a subset of participants who underwent FDG-PET, PiB-PET, MRI, and cognitive testing at the current time point. The participants also underwent structural MRI scanning 10 years prior and cognitive testing 5 and 10 years prior to the current investigation. In total, 82 participants were included in the study. Of those, 65 participants underwent both FDG-PET and PiB-PET scanning. Additional five subjects only completed the PiB-PET, and 12 only completed the FDG scan. All subjects completed both MRI scans. Participant characteristics, neuropsychological outcomes, and PET data are presented in Table 1. The study setup is summarized in Fig. 1.

**Table 1:** Participant demographics

Participants, *n*	82	
Age, years [sd] {range}	67.2 [0.6] {66–68}	
Education years, mean [sd]	10.6 [1.7]	
Amyloid positivity,* n* (%)	15 (21%)	
Fazekas score:		
Deep white matter hyperintensity, (frequency)	0 (4), 1 (75), 1,5 (1), 2 (1) 3 (1)	
Periventricular hyperintensity, (frequency)	1 (67), 1.5 (5), 2 (9), 3 (1)	
PiB-PET SUVr, mean [sd]		
Mean cortical	1.24 [0.16]	
Precuneus	1.33 [0.24]	
PCC	1.37 [0.23]	
ICC	1.37 [0.17]	
CACC	1.34 [0.24]	
Neuropsychological testing:	At age ≈ 67	At age ≈ 63
ACE (# correct) [sd]	95.8 [3.4]	93.6 [4.7]
IST (# correct) [sd]	33.5 [10.2]	32.2 [10.1]
VPA Word pairs (# errors) [sd]	10.1 [7.8]	12.2 [7.9]
VPA Retention (# errors) [sd]	3.6 [3.0]	5.9 [3.3]
PAL (# errors, 8 trials) [sd]	11.4 [14.2]	11.2 [13.9]
SDMT (# correct) [sd]	46.9 [8.9]	48.0 [8.6]
MRI volumetry:	At age ≈ 67:	At age ≈ 57:
Total brain volume (ml) [sd]	1142.7 [92.2]	1185.3 [89.2]
Total cortical gray matter volume (ml) [sd]	484.1 [36.2]	487.3 [34.4]
Total gray matter volume (ml) [sd]	642.8 [43.5]	652.5 [40.6]
Lateral ventricle (ml) [sd]	18.5 [8.0]	12.9 [5.0]
Precuneus cortical thickness (mm) [sd]	2.31 [0.09]	2.32 [0.10]
PCC cortical thickness (mm) [sd]	2.29 [0.10]	2.37 [0.11]
ICC cortical thickness (mm) [sd]	2.16 [0.13]	2.27 [0.15]
CACC cortical thickness (mm) [sd]	2.31 [0.13]	2.38 [0.15]

*PCC* posterior cingulate cortex, *ICC* isthmus cingulate cortex, *CACC* caudal anterior cingulate cortex, *ACE* Addenbrook’s Cognitive Examination, *IST* Intelligenz Struktur Test, *VPA* Verbal Paired Associates, *PAL* Paired Associative Learning, *SDMT* Symbol Digit Modalities

### Standard protocol approvals, registrations, and patient consents

This study was approved by the Capital Region of Denmark’s Committee on Health Research Ethics (H-1–2014032) and conducted according to the Declaration of Helsinki. All participants provided written consent regarding their participation and publication of the current data.

#### Neuropsychological examination

Cognitive functions were assessed using the following neuropsychological tests: Addenbrooke’s Cognitive Examination (ACE) to rule out dementia or MCI disease. Verbal Paired Associates (VPA) (word pairs and retention) to measure verbal memory functions. Symbol Digits Modalities Test (SDMT) to measure processing speed. Subtests from Intelligenz-Struktur Test (IST) to estimate intelligence. The Paired Associative Learning (PAL) test from the Cambridge Neuropsychological Test Automated Battery (CANTAB) was administered to measure visual associative memory. We used PAL 8 Shapes Total Errors (adjusted) as variables from the PAL test, where we utilized the current test score and the change in scores between ages 62 and 67. PAL 8 Shapes, due to its difficulty, is less likely to be affected by ceiling effects and is regarded as particularly suited for measuring memory in high-functioning individuals [22]. All neuropsychological tests were administered by certified hospital staff.

#### MRI image acquisition

MRI was performed on a Philips Achieva 3 T scanner (Philips, Best, The Netherlands) with a 32-channel phased array head coil. Whole-brain 3D T1-weighted high-resolution anatomical scans were acquired using a turbo field echo sequence (echo time (TE) = 5.11 ms; repetition time (TR) = 11.2 ms; flip angle = 8^◦^, field of view (FOV) = 240 × 256 × 180 mm^3^; voxel size = 0.70 × 0.76 × 0.70 mm^3^). Images were segmented based on the Desikan-Killiany atlas using the standard longitudinal segmentation pipeline from the FreeSurfer software suite (v7.1.1, Martinos Center for Biomedical Imaging, MA, USA).

#### PET image acquisition

The participants were scanned on a Siemens Biograph Vision 600 PET/CT scanner (Siemens Healthcare). The cerebral A*β* accumulation was examined using the PiB radiotracer, and the relative cerebral glucose consumption was assessed using FDG as the radiotracer according to recent guidelines [23]. PiB images were measured by a 20-min static acquisition initiated 40 min after injection of approximately 250 MBq PiB. For the FDG imaging, a static 10-min PET frame was acquired 45 min after injection of approximately 200 MBq FDG. The PiB data were reconstructed using a 3D Ordered Subset Expectation Maximization (OSEM) algorithm with four iterations, five subsets, and 5 mm Gaussian reconstruction filters. The FDG data were reconstructed with 12 iterations, five subsets, and 3 mm Gaussian reconstruction filters. Both PiB and FDG were reconstructed into images with 440 × 440 in-plane matrix size and 159 slices, resulting in a voxel size of 0.825 × 0.825 × 1.6 mm^3^. A low-dose radiation CT scan (120 kV, 30 mAs, 3-mm slice thickness, 512 × 512 image matrix size, voxel size 0.59 × 0.59 mm^2^, H19s convolution kernel) was acquired before each PET scan for attenuation correction. The PET data were further corrected for randoms, scatter, decay, and dead time. The reconstructed PET images were co-registered with the anatomical MRI images using the FreeSurfer Software Package to extract PET values from brain regions segmented from the MRI images.

#### Statistical analysis

**Fig. 2:**
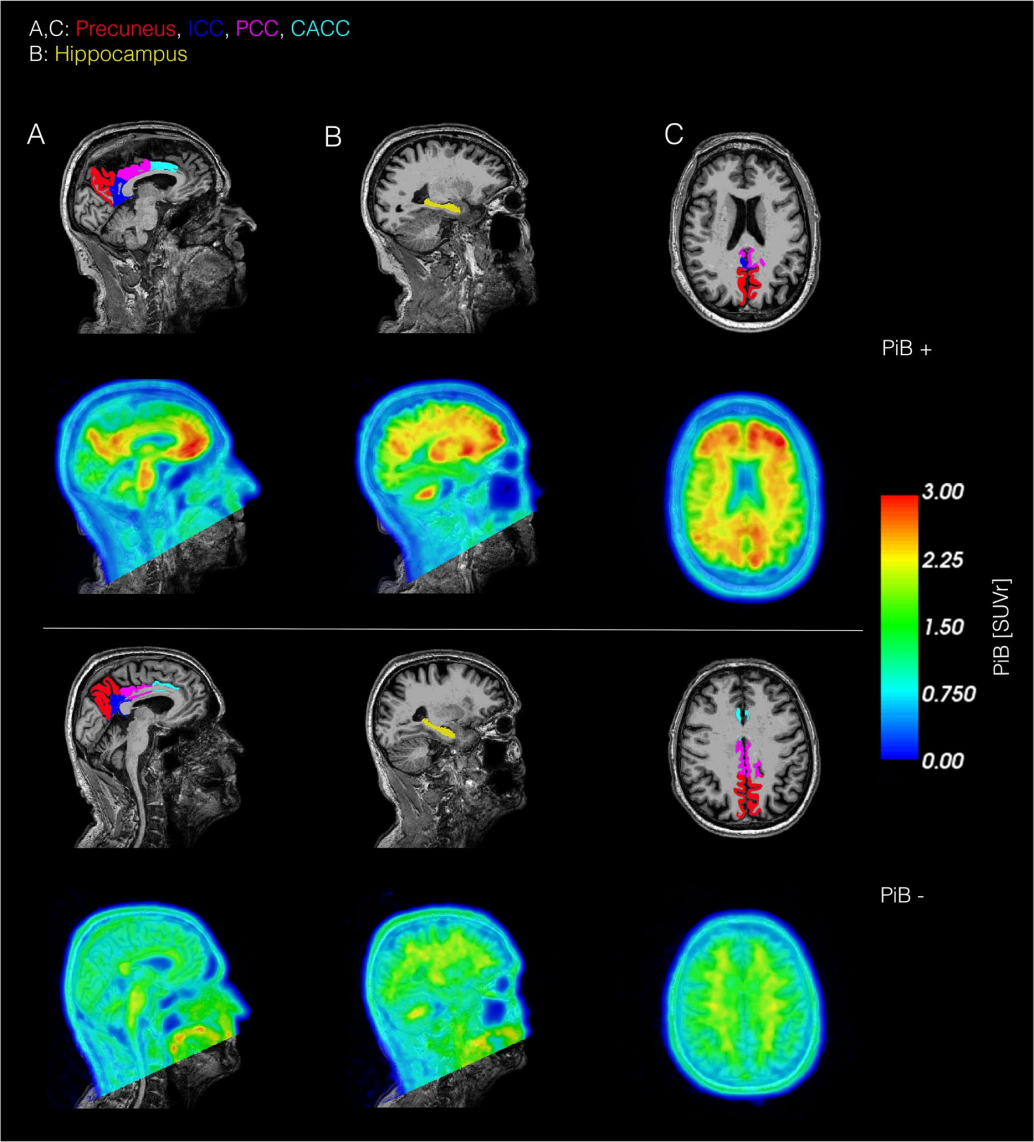
Illustration of brain regions examined in the PiB AD-ROI and hippocampus and examples of corresponding PiB SUVr images in two representative subjects. The included brain were the precuneus, isthmus cingulate cortex, posterior cingulate cortex, and caudal anterior cingulate cortex (all demonstrated in **A** and **C**. The hippocampus is not included in the AD-ROI, but is illustrated in panel B. The top row shows a PiB-positive subject, and the bottom row shows an PiB-negative subject. A*β* accumulation is clearly visible in the PiB positive subject in regions belonging to the AD-ROI. PCC; posterior cingulate cortex, ICC; isthmus cingulate cortex, CACC; caudal anterior cingulate cortex

**Fig. 3:**
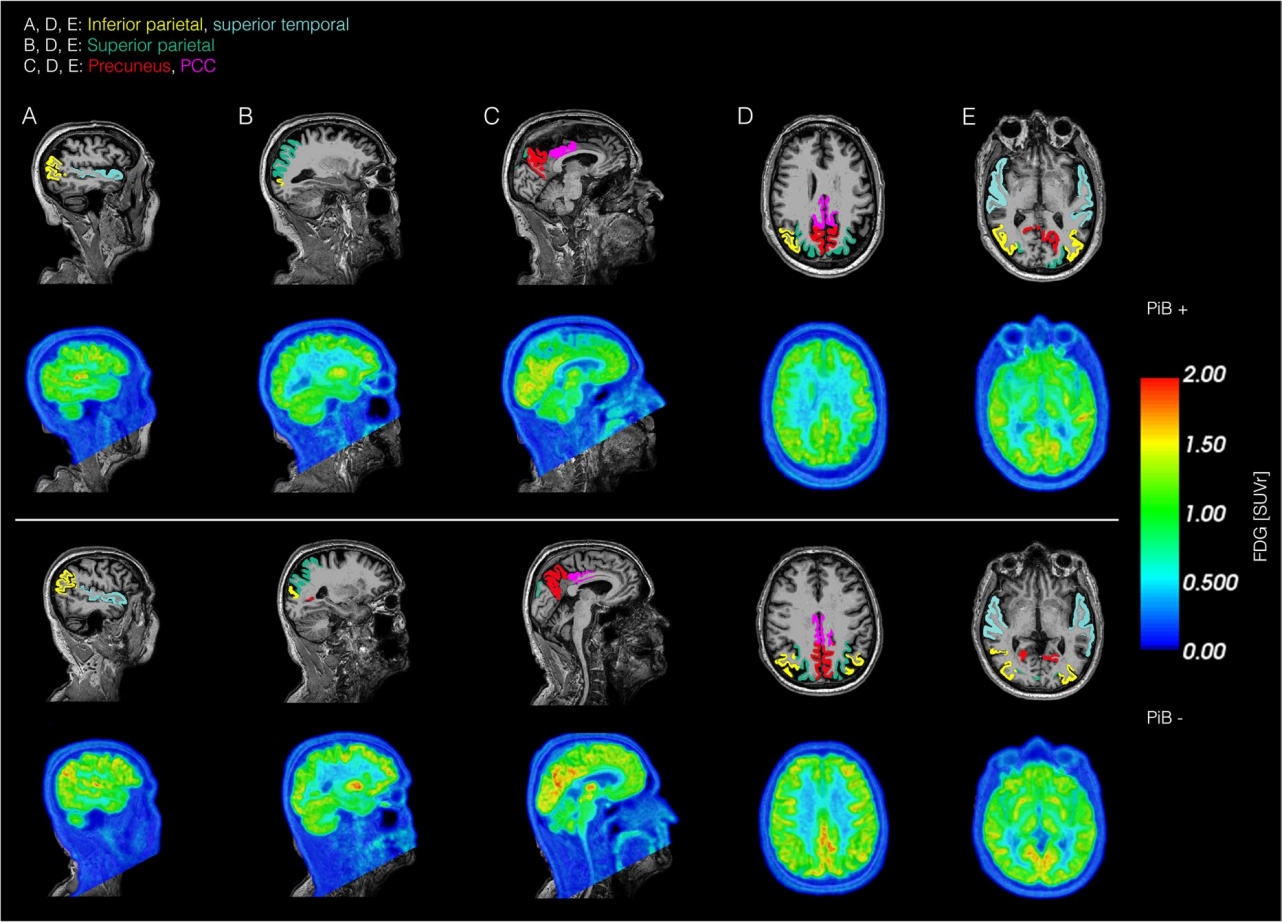
Illustration of brain regions examined in the FDG AD-ROI and examples of corresponding FDG SUVr images in two representative subjects. The included regions were the inferior parietal and superior parietal cortex (demonstrated in **A**, **D,** and **E**), superior parietal cortex (**B**, **D**, **E**), precuneus, and posterior cingulate cortex (**C**, **D**, and **E**). The top row shows FDG SUVr in a PiB positive subject, and the bottom row shows an PiB-negative subject. PCC; posterior cingulate cortex

Correlations between cognition and A*β* accumulation or glucose metabolism were assessed using a linear regression model with the cognitive score as the dependent value and standardized uptake value ratio (SUVr) of PiB or FDG as the independent value. SUVr values were extracted from two regions of interest (ROI) typically affected by A*β* deposition or hypometabolism in AD and are referred to as the AD-ROI. For PiB-PET, the AD-ROI included the following regions: precuneus, posterior cingulate cortex (PCC), isthmus cingulate cortex (ICC), and caudal anterior cingulate cortex (CACC) (Fig. 2). For FDG-PET, the AD-ROI included the following regions: the superior parietal cortex, inferior parietal cortex, superior temporal cortex, precuneus, and PCC (Fig. 3). The PET images were co-registered with the anatomical MRI images using FreeSurfer. The brain segmentation from the MRI images was used to extract mean cortical SUVr values in the regions defined by the AD-ROIs. The mean SUVr values were calculated as weighted means based on the volume of the included regions. The cerebellar cortex without vermis was used as the reference region for both FDG and PiB SUVr. We similarly extracted cortical thickness/volume in the AD ROIs as the weighted mean, similar to the SUVr values.

The regression models were calculated using R (R Core Team, Vienna, Austria). Multiple linear regression models assessed the association between the SUVr in the AD-ROIs and cognitive score. Cortical thickness in the corresponding ROIs was added as a covariate to control for potential partial volume effects and atrophy. PAL 8 shapes scores were log-transformed (constant of 1 added because of 0 values) before entering the statistics to correct for right-skewed distributions. The decline in the PAL scores was calculated by subtracting the log-transformed score obtained at 63 years from that at 68 years. A negative number indicates declining performance since the PAL measures the number of errors.

If SUVr values in the AD-ROI correlated with cognition, we conducted a regional surface-based analysis between SUVr (PiB or FDG) and the relevant test. This was done for the association between PiB SUVr with PAL 8 Shapes and FDG SUVr with VPA Retention. The surface-based analysis was performed using the standard pipeline from the FreeSurfer software suite. The cortical PiB SUVr was projected onto the pial surface based on the segmentation of the MRI anatomical images, and linear regression models were calculated for each surface vertex. The analysis was corrected for multiple comparisons using the Freesurfer precomputed Monte Carlo simulation with cluster-forming thresholds of *p* < 0.01 and cluster-wise *p* values < 0.05.

Finally, linear regression was used to assess the relationship between mean global cortical PiB SUVr and global measures of brain atrophy, defined as atrophy rate of total brain volume, total cerebral cortical volume, and total gray matter volume. The atrophy rate was calculated as a percentage volume decline between the current MRI scans and scans from 10 years ago. The correlation between PiB SUVr and FDG SUVr in the AD-ROI was tested using a linear regression model.

## Results

### Missing/removed data

For one subject, the FDG SUVr in the AD-ROI was a significant outlier (< − 5 SD) and was removed from further analysis. There was one missing data point for IST and three for PAL due to the participants not completing the cognitive testing. There were three missing data points for brain atrophy due to failed segmentation. Six of the current participants did not partake in the previous examination 5 years prior, and additionally, one VPA Retention was missing at this time due to scheduling delays. Two of the participants from the current examination had not participated in the examination 10 years prior.

### Aβ deposition and brain atrophy

**Fig. 4:**
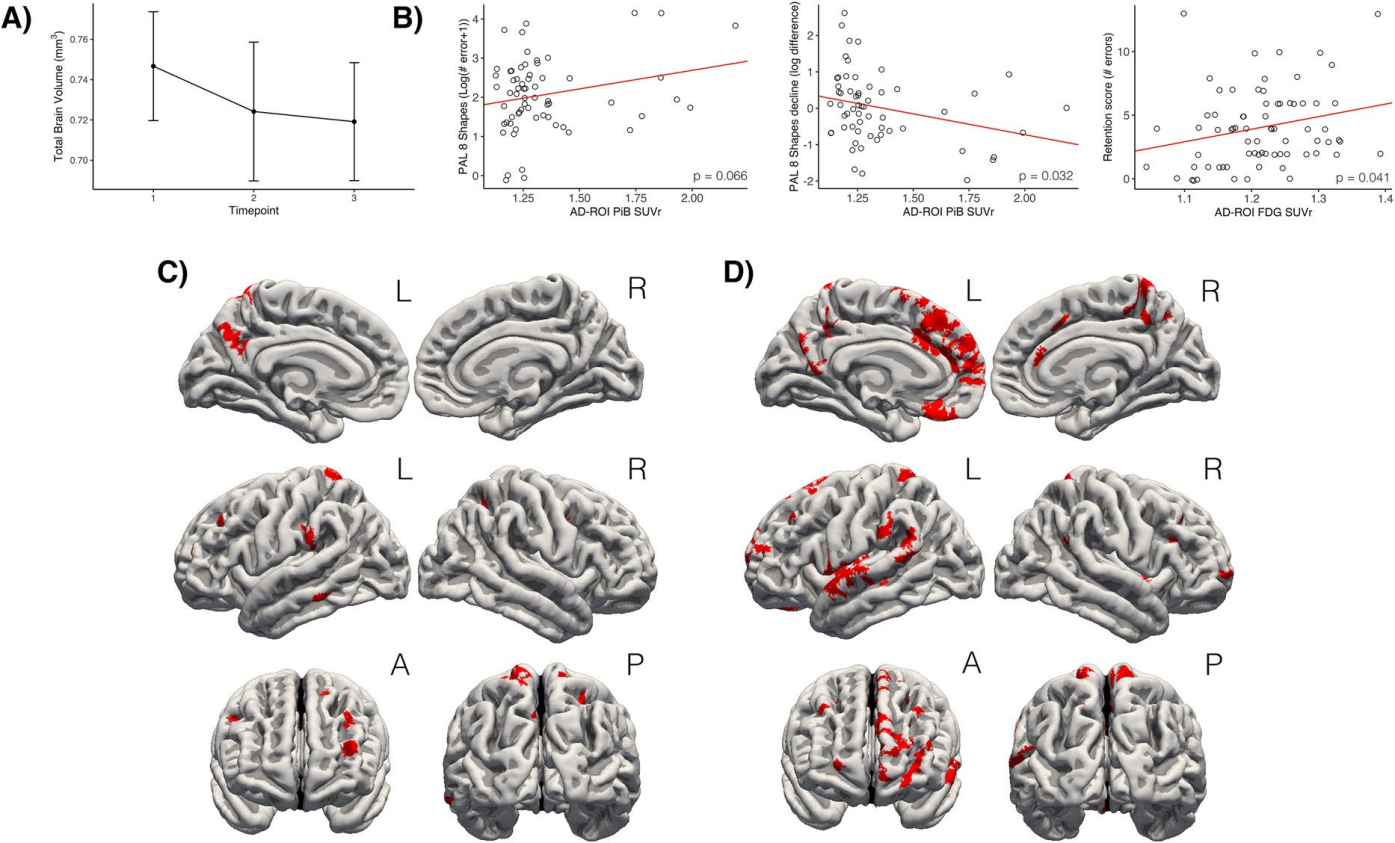
Summary of main findings. **A** Trajectory of total brain volume atrophy in a 10-year period. **B** Plots illustrating the correlation between cognition and PET SUVr. The first two scatterplots show that worse performance and increased decline in PAL 8 Shapes are associated with higher PiB SUVr in the AD-ROI. Similarly, the third scatterplot shows how worse performance on VPA Retention is correlated with higher FDG SUVr in the AD-ROI. **C** Results from the surface-based cortical analysis, showing regions where worse PAL 8 Shapes performance is associated with increased PiB SUVr. **D** Results from the surface-based cortical analysis, showing regions where increased decline in PAL 8 Shapes performance is associated with increased PiB SUVr

We observed a reduction in measures of total brain volume between the current MRI scans and those acquired 10 and 5 years ago (Fig. 4a). On average, total brain volume had an annual atrophy rate of 0.36%, total cortical volume of 0.06%, and total gray matter volume of 0.15%. The observed brain volume loss was as expected according to previous literature [24, 25]. However, we observed no significant correlations between global atrophy rate in total brain volume, total cortical volume, or total gray matter volume with mean cortical PiB SUVr (See Supplementary Figure S1). Overall, these results indicate that the A*β* accumulation observed in this cohort had not caused accelerated atrophy of the brain.

### Aβ and cognition

None of the ACE test scores were below the recognized cut-off score of 82/83 [26], meaning that all the participants demonstrated clinically normal cognitive functioning. The cognition results are summarized in Table 1. We observed a borderline significant correlation between PiB SUVr and worse performance on PAL (*p* = 0.066) and a significant correlation between increased PiB SUVr and more decline in PAL score (*p* = 0.032) (Table 2), demonstrating that participants who did not maintain cognitive performance had increased A*β* deposition (Fig. 4b) (Table 2). The associations between PiB SUVr and cognition did not remain significant after correcting for multiple testing. We did not observe significant correlations with the other cognitive parameters (Supplementary Table 1).

**Table 2:** Results from multiple linear regression models

		Dependent variable	
	Model 1	Model 2	Model 3
	Log(PAL 8 Shapes + 1)	Log(PAL decline + 1)	VPA Retention
PiB SUVr	0.950	− 1.145*	
	*p* = 0.066	*p* = 0.032	
FDG SUVr			9.874*
			*p* = 0.041
Cortical thickness	0.830	− 0.689	0.222
	*p* = 0.607	*p* = 0.689	p = 0.967
Observations	64	59	73
R^2^	0.063	0.086	0.059
Adjusted R^2^	0.032	0.054	0.032
Residual Std. Error	0.925 (df = 61)	0.942 (df = 56)	2.976 (df = 70)
F Statistic	2.038 (df = 2; 61)	2.648* (df = 2; 56)	2.195 (df = 2; 70)

*p < 0.05

We proceeded to do a regional surface-based analysis to examine which specific brain areas demonstrated a correlation between a decline in PAL score and higher PiB SUVr. This analysis yielded significant correlations in multiple clusters located in the frontal (*p* < 0.042), temporal (*p* < 0.016), parietal cortex (*p* < 0.039), precuneus (*p* < 0.040), and cingulate cortex (*p* < 0.013), after correcting for multiple comparisons. Similarly, we observed a significant correlation between the current PAL score and PiB SUVr in clusters located in the temporal (*p* < 0.001), parietal (p < 0.030), and frontal cortex (*p* < 0.036), precuneus (*p* < 0.041), and cingulate cortex (*p* < 0.01) after correction of multiple comparisons. The affected regions correspond to regions typically afflicted by AD (Fig. 4c, d).

### Higher parieto-temporal FDG SUVr is associated with a worse memory outcome

We observed a significant correlation between worse VPA Retention score and higher FDG SUVr in the AD-ROI (*p* = 0.040), indicating that higher cerebral glucose metabolism is correlated with worse cognitive performance (Fig. 4b and Table 2), but this did not remain significant after correcting for multiple testing. We did not observe significant correlations between FDG SUVr and other cognitive parameters. Regional surface-based analysis between FDG SUVr, and VPA retention score did not reveal any significant correlations after correcting for multiple testing.

### No correlation between FDG and PiB

There were no significant associations between FDG and PiB SUVr in the AD-ROI (*p* = 0.982).

## Discussion

### Aβ deposition in AD-critical areas is associated with worse memory performance but not associated with an accelerated atrophy rate

The current study observed a significant association between worse visual memory performance and greater visual memory decline with increased A*β* deposition in known AD-critical areas. However, A*β* deposition was not associated with accelerated brain atrophy. These findings suggest that A*β* deposition in AD critical areas affects memory function and may provoke cognitive decline before accelerated atrophy, perhaps by compromising optimal neuronal functioning. This finding is concordant with a comprehensive study by Wang et al. [27] who observed memory functions to be affected prior to hypometabolism and accelerated hippocampal atrophy in preclinical AD. Other studies have also found an association between increased A*β* deposition and worse performance in global cognition, memory, and executive functions [28, 29]. Conversely, some longitudinal studies have diverged by revealing a simultaneous acceleration in brain atrophy alongside greater cognitive decline, highlighting the intricate nature of this phenomenon [28, 30].

To address the apparent discrepancies between our study and those conducted by Donohue et al. (2017) [30] and Petersen et al. (2016) [28], several key distinctions should be considered. First, the age of the participants in those studies was notably higher, ranging from 73 to 80, in contrast to our cohort of 68 years. Second, these studies reported higher average PiB SUVr values, consistent with increased PiB SUVr with age. Combined with our results, this suggests that A*β* may be associated with accelerated atrophy but not necessarily in the initial accumulation phase. Instead, it may manifest as an effect observed after more severe deposition and over prolonged periods. Furthermore, differences in the operationalization of atrophy rates between our studies could also contribute to the varying results. While the prior studies relied on ventricular volume to measure brain atrophy, we assessed atrophy across a wide selection of brain regions. Additionally, the intervals between examinations differed, with the earlier studies employing shorter intervals of 3–8 years compared to our extended 10-year interval. Lastly, the aforementioned studies first measured PiB deposition and, subsequently, atrophy. In contrast, we measured the decennial atrophy rate before PiB deposition, making it challenging to determine when our participants became PiB positive.

### Hypermetabolism in AD-critical areas is associated with worse memory performance

We found that increased FDG SUVr in the AD-susceptible regions was significantly associated with worse memory performance. Initially, this finding may seem counter-intuitive, as events associated with AD are A*β* deposition, glucose hypometabolism, and cerebral atrophy [15]. Specifically, FDG-PET hypometabolism is consistently observed in temporoparietal regions in AD and MCI [31, 32]. However, the specific patterns of change in cerebral metabolism concerning AD and how they differ before and after conversion are still unclear. Whereas many studies report reduced glucose metabolism, other studies find increases in cerebral glucose metabolism [33–38].

Hypermetabolism may occur as a reaction to initial A*β* deposition. In support of this, studies have found associations between increased glucose metabolism and A*β* deposition in regions affected by AD in both MCI patients [39] and healthy controls [34]. However, other studies indicate that hypermetabolism in AD regions in MCI and at-risk controls may be more linked to tau accumulation or the interactional effect of A*β* and tau in the brain. For instance, Rubinski et al. (2020) [33] found that higher tau-PET was associated with higher FDG-PET in multiple regions within the temporoparietal and cingulate cortices in amyloid-negative MCI subjects. In contrast, the opposite pattern was observed for A*β* positive subjects. They also observed a significant association between increased FDG-PET metabolism in the right middle frontal gyrus and lower memory scores. This finding is in accordance with other studies, which have also observed increased tau-PET associated with increased FDG-PET in cognitively normal participants with low levels of A*β* deposition [37, 38]. Thus, the observed hypermetabolism may be more linked to tau than A*β* and may be a precursor to the later-observed hypometabolism, which occurs when the amyloid burden has accumulated beyond the capacity thresholds of the brain [36].

These findings are coherent with functional MRI (fMRI) studies, which show hyperactivity in temporoparietal regions of subjects with autosomal AD and MCI [40–42], and for this hyperactivity to be related to cognitive decline and increased atrophy rate [43]. Other fMRI studies have also reported significant associations between A*β* and tau accumulation with increased activity in the medial temporal lobes [44, 45]. The increased brain activity in prodromal AD may reflect compensatory neural recruitment to maintain normal cognitive functions in the early pathological stages. Alternatively, the increased activity may also reflect aberrant neural activity or excitotoxic disease processes [41, 42, 44], such as altered ionic homeostasis caused by the excessive inflow of calcium into neurons [46] or disruption of GABAergic neuronal networks [47].

### Relationship between Aβ deposition and glucose metabolism

Since AD pathology follows a temporal ordering with probable downstream effects, the relationship observed between PiB and FDG depends on when it is assessed. In patients with AD, studies typically report that increased A*β* deposition is associated with hypometabolism [39]. This is likely due to downstream pathological effects from amyloidosis, which has caused neuronal damage or cerebrovascular dysfunction [48]. In contrast, the opposite pattern is sometimes observed between PiB and FDG in MCI and normal controls, where studies have observed increased FDG in response to A*β* or tau accumulation [33–36, 39]. This is believed to reflect a compensatory mechanism, where A*β* or tau accumulation has led to disruption of neural functioning, which the brain attempts to compensate for with increased neuronal activity. Alternatively, a higher metabolic rate may contribute to accelerated A*β* deposition [39]. In support of this, Benzinger et al. (2013) [35] observed hypermetabolism in regions that are typically found to suffer from hypometabolism in AD (i.e., lateral parietal cortex, precuneus, posterior cingulate cortex) in autosomal dominant AD mutation carriers approximately 25 years before estimated age of AD onset, compared to normal controls. Thus, the lack of correlation between PiB and FDG in the current study may be because the A*β* deposition is insufficient to affect glucose metabolism, which is in line with the age and health of the current participants. However, considering the temporal ordering of AD biomarkers [15] and the amyloid cascade hypothesis, we deem it likely that the A*β*-positive subjects in our study are experiencing the first pathological ’hit.’ Thus, we predict their subsequent changes will be hypermetabolism, hypometabolism, and accelerated atrophy. Furthermore, in future studies, it will be interesting to investigate whether the participants with current hypermetabolism will have increased A*β* deposition compared to the rest of the group.

## Strengths and limitations

A primary strength of the study is the acquisition of longitudinal brain structure and cognition data spanning up to 10 years, allowing us to assess associations between A*β* accumulation and cerebral glucose metabolism with age-related changes in brain structure and cognition.

One study limitation is the relatively small sample size relative to the effect sizes. This could contribute to the associations between SUVr in the AD-ROI and cognition not surviving multiple testing corrections. However, although the findings from the current study are preliminary, we hold that they are valuable for two main reasons. First, our results indicate that while the associations between PiB SUVr in the AD-ROI and cognition did not withstand multiple comparisons, the persistent significance of the surface-based analysis suggests a possible benefit of exploring individual regions rather than an ROI-based approach. Second, this study possesses valuable longitudinal brain structure data spanning 10 years, allowing us to assess associations between SUVr and age-related changes in volume. Hence, we consider this a meaningful study, which we hope will motivate future research to delve into this topic and challenge the existing notions on the temporal orderings of AD biomarkers.

As previously discussed, the current study’s generalizability is constrained because it utilizes an all-male cohort [19]. This is because when the Metropolit cohort was established in 1965, its primary goal was to examine socioeconomic mobility. Only men were included in the cohort since social status and mobility at the time were predominantly associated with the job of the male breadwinner [18]. Nevertheless, we consider the data collected over the years useful for studying aging and feel they should be employed despite the apparent constraints of analyzing a male-only cohort. An additional limitation is potential attrition effects, indicating that the participants in the worst physical and mental condition may be less likely to return for follow-up examinations.

Since PET imaging was only conducted at one point, we could not determine the trajectory of these variables or examine the causal relationship of PiB accumulation with cognition and atrophy rate. However, it is well grounded in the literature that A*β* deposition is one of the first pathological markers of AD, accumulating over many years before plateauing. Thus, considering the demographic of our cohort, we assume that participants who currently exhibit heightened A*β* deposition also had heightened A*β* deposition relative to the rest of the group 10 years ago. In light of this assumption, we proposed that PiB SUVr would be correlated with cognition and atrophy.

Another limitation is that tau accumulation was not examined in the current project. Ossenkoppele et al. (2022) found a reinforced effect of tau accumulation on the steeper cognitive decline observed in A*β*-positive individuals [49]. This effect showed that tau, particularly in the temporal neocortical region, was associated with steeper cognitive decline and a higher risk of development of MCI and dementia than only A*β*-positivity. This indicates elevated A*β* is a risk factor for subsequent cognitive decline and AD pathology. However, tau accumulation may constitute an additional risk that may further propagate a pathological trajectory.

## Conclusion

Our data indicate that A*β* may lead to subtle memory deficits and cognitive decline before observable associations with abnormal cerebral atrophy or hypometabolism. These results suggest that A*β* might affect neuronal functioning before accelerated atrophy occurs. Moreover, our results indicate that cerebral hypermetabolism is associated with worse cognitive performance, perhaps due to early pathological effects. These findings provide insight into the temporal ordering of AD biomarkers and possible mechanisms behind memory deficits, constituting a hallmark symptom of AD.

### Supplementary Information

Below is the link to the electronic supplementary material.

Supplementary file1 Results from multiple linear regression models (DOCX 1.70 MB)

Supplementary file2 Linear associations between global cortical PiB SUVr and decennial atrophy of total brain volume, cortical volume, and total gray matter volume. There were no significant associations between PiB SUVr and any of the measures of cerebral atrophy rate (TIF 9342 KB)

## Data Availability

Data is not publicly available due to ethical restrictions and participant privacy concerns. Requests for access to the data may be submitted to Prof. Martin Lauritzen for review and approval.
