# Supplementary material for: Early cerebral amyloid-β accumulation and hypermetabolism are associated with subtle cognitive deficits before accelerated cerebral atrophy
Source: GeroScience. 2023 Dec 16;46(1):769–82. doi: 10.1007/s11357-023-01031-w (PMC10828321; doi:10.1007/s11357-023-01031-w)
Supplement: Supplementary file 1 — Supplementary file1 Results from multiple linear regression models (DOCX 1.70 MB) [file 11357_2023_1031_MOESM1_ESM.docx]

Supplementary information

| PiB AD-ROI |  |  |  | FDG AD-ROI |  |  |  |
| --- | --- | --- | --- | --- | --- | --- | --- |
| IST (# correct) | -0.807 | -0.023 | 0.884 | IST (# correct) | -15.364 | -0.017 | 0.337 |
| VPA Word pair (# errors) | 0.0431 | -0.025 | 0.919 | VPA Word pair (# errors) | 16.97 | 0.010 | 0.186 |
| VPA Retention (# errors) | 0.551 | -0.029 | 0.740 | VPA Retention (# errors) | 9.874 | 0.032 | 0.040 |
| PAL 8 Shapes (# errors) | 0.950 | 0.0318 | 0.065 | PAL 8 Shapes (# errors) | 0.763 | 0.026 | 0.603 |
| SDMT (# correct) | -0.632 | -0.018 | 0.894 | SDMT (# correct) | -5.127 | -0.026 | 0.709 |

**Supplementary Table 1.** Results from multiple linear regression models.


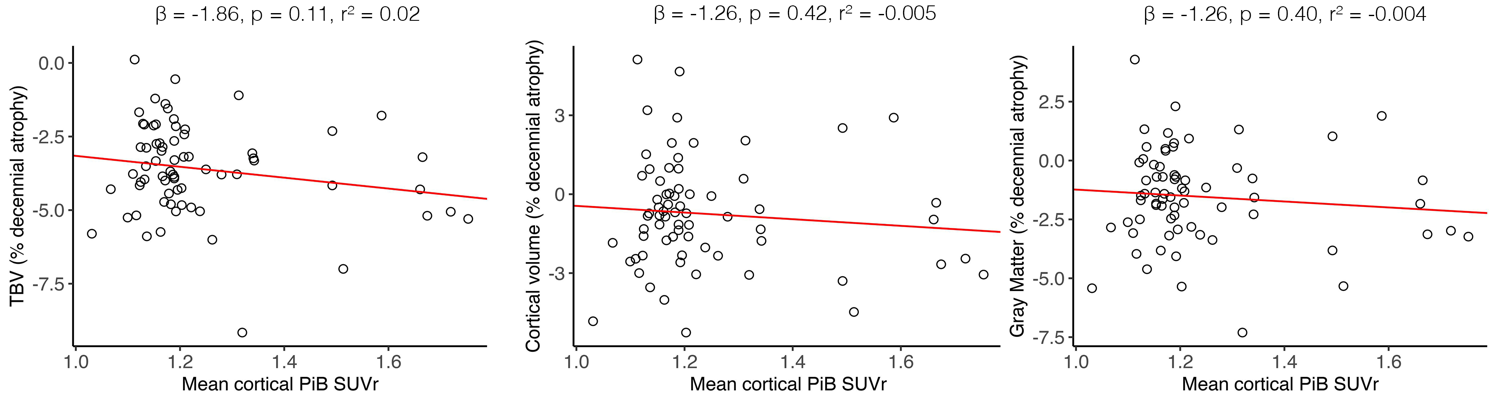


**Supplementary Figure 1**. Linear associations between global cortical PiB SUVr and decennial atrophy of total brain volume, cortical volume, and total gray matter volume. There were no significant associations between PiB SUVr and any of the measures of cerebral atrophy rate.
