# Supplementary figures and images for: Early cerebral amyloid-β accumulation and hypermetabolism are associated with subtle cognitive deficits before accelerated cerebral atrophy
Source: GeroScience. 2023 Dec 16;46(1):769–82. doi: 10.1007/s11357-023-01031-w (PMC10828321; doi:10.1007/s11357-023-01031-w)

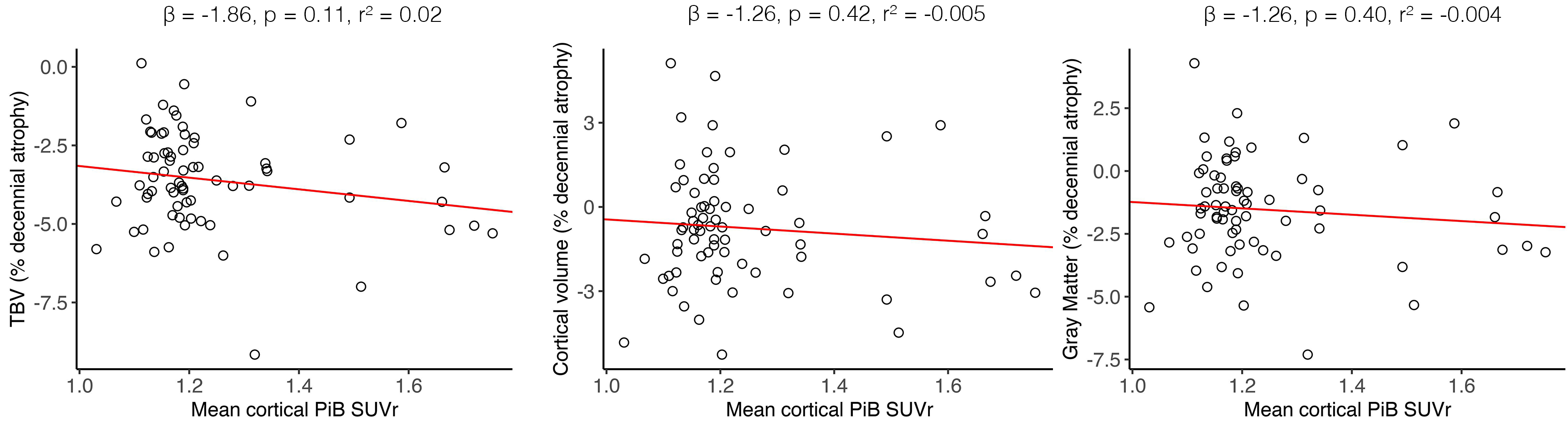

Supplement: Supplementary file 2 — Supplementary file2 Linear associations between global cortical PiB SUVr and decennial atrophy of total brain volume, cortical volume, and total gray matter volume. There were no significant associations between PiB SUVr and any of the measures of cerebral atrophy rate (TIF 9342 KB) [file 11357_2023_1031_MOESM2_ESM.tif]
